# Health economic evaluation of microprocessor and non-microprocessor controlled prosthetic knees

**DOI:** 10.33137/cpoj.v8i2.45823

**Published:** 2025-10-30

**Authors:** C.E. Bosman, C.K. van der Sluis, A.H. Vrieling, J.H.B. Geertzen, B.L. Seves, H. Groen

**Affiliations:** 1 Department of Rehabilitation Medicine, University of Groningen, University Medical Center Groningen, Groningen, The Netherlands.; 2 Department of Epidemiology, University of Groningen, University Medical Center Groningen, Groningen, The Netherlands.

**Keywords:** Lower Limb, Amputation, Prostheses, Cost Analysis, Quality of Life, Questionnaire, Mobility, Microprocessor Knee, Cost-effectiveness, Knee Disarticulation, Transfemoral

## Abstract

**BACKGROUND:**

Use of a microprocessor-controlled knee (MPK) compared to a non-microprocessor-controlled knee (NMPK) can lead to improved walking ability, confidence and satisfaction. However, the MPK is more expensive than the NMPK and it is unknown whether the higher costs outweigh the potential benefits.

**OBJECTIVE:**

To evaluate the cost-utility and cost-effectiveness of MPKs and NMPKs from a societal perspective in the Netherlands.

**METHODOLOGY:**

Participants completed the Dutch version of the EuroQol - five dimensions - five levels (EQ-5D-5L) to assess health-related quality of life, three subscales (ambulation, utility and well-being) of the Prosthesis Evaluation Questionnaire (PEQ) to assess prosthesis-related quality of life and a cost-questionnaire from societal perspective. Incremental cost-utility ratio (ICUR) and incremental cost-effectiveness ratio (ICER) were calculated and the ICUR was compared with the Dutch willingness-to-pay threshold. Bootstrapping was used to estimate statistical uncertainty, and multiple imputation was applied to account for missing values.

**FINDINGS:**

In total, 111 participants were included (37 female, 73 male, 1 unknown; 71 transfemoral, 39 knee disarticulation, 1 unknown; age 64 ± 13 years; 49 NMPK users, 62 MPK users). The cost-utility analysis demonstrated that the MPK yielded an increase of 0.032 quality adjusted life years (QALY) but at considerably higher costs. The mean cost difference was € 14,626, resulting in a mean ICUR of € 457,063 per QALY gained. The cost difference was mainly driven by acquisition costs but was partially compensated by lower costs of work absence, health care consumption and household care.

**CONCLUSION:**

The cost-effectiveness analyses demonstrated that the MPK is likely to provide benefits in term of prosthesis-specific quality of life, but at higher costs. However, short-term (6 months) improvement in health-related quality of life was too small to result in substantial QALY gain to offset the higher costs of the MPK and result in an incremental cost-utility ratio below the generally accepted willingness-to-pay thresholds.

## INTRODUCTION

A lower limb amputation (LLA) can negatively impact daily activities, participation, and other aspects of life.^1-3^ Individuals with a transfemoral amputation or knee-disarticulation can use a prosthesis with a knee unit. Prostheses for persons with a transfemoral amputation or knee disarticulation feature knee units, which are typically classified as non-microprocessor controlled (mechanical) knees (NMPK) or microprocessor controlled knees (MPK). NMPKs may be purely mechanical or can include pneumatic or hydraulic systems to assist in swing and/or stance phase control. MPKs, equipped with sensors and a microprocessor, can automatically adjust to the user's movements during swing and stance phases. The higher acquisition cost of the MPK is due to not only the inclusion of more advanced and expensive electronic components, but also research and development expenses, as well as testing and regulatory compliance requirements. Nevertheless, research suggests that the MPK may offer added value for both active individuals, due to their adaptive capabilities,^4^ and older users, for whom safety and stability are particularly beneficial.^5^ Despite these potential benefits of the MPK, such as reduced stumbles and falls,^6-10^ and improvements in walking speed, satisfaction, confidence and quality of life (QoL),^6,8,9,11-13^ it is unclear whether these benefits justify the additional expense.

In the Netherlands, approximately 10,000 people use a lower limb prosthesis (LLP),^14^ with an estimated one-third having a transfemoral amputation or knee disarticulation.^15^ Over the past decade, annual healthcare costs for LLPs have increased by over 30%, from €30.4 million in 2012 to €42.5 million in 2019*, with per-user costs rising from €3,110 to €4,400.^14^ The relationship between these cost increases and MPK prescriptions has not been studied. Besides the prosthesis acquisition cost, other factors such as visits to healthcare professionals, prosthesis repairs and home environment adjustments must be considered. Additionally, MPK use can affect a person's productivity and ability to contribute to society. To fully understand the cost-utility and cost-effectiveness of prosthetic knees, a societal perspective that includes all these factors is essential.

Economic evaluations can be used to inform policy decisions about the allocation of health care funds irrespective of the disease.^16^ The most commonly used types of economic evaluations are the cost-utility analysis and cost-effectiveness analysis.^17^ For both the cost-utility analysis and cost-effectiveness analysis, the difference in costs (incremental costs) is divided by the difference in effects (incremental effects), resulting in either an incremental cost utility ratio (ICUR) or an incremental cost effectiveness ratio (ICER).^17^

In a cost-utility analysis, the effects are expressed in quality-adjusted life years (QALYs), and this analysis can therefore be used for broad comparisons. The value of the ICUR can be compared across different diseases and to the threshold value for willingness-to-pay for one QALY. The cost-effectiveness analysis on the other hand, can use prosthesis-related effect measures for a more specific comparison.

Several studies have performed a cost-utility analysis to compare the MPK to the NMPK, and reported ICURs within the willingness-to-pay threshold.^18-22^ However, ICURs varied widely,^18,20^ likely due to differences in study design and perspectives. Variations in healthcare costs and insurance coverage across countries also contribute to this disparity, raising questions about the generalizability in the Netherlands. A Dutch study by Seelen et al.^23^ compared MPKs to NMPKs in a cost-consequence design and reported that the average annual costs per person was lower for MPK users compared to NMPK users, but without calculating incremental cost and effect differences. Higher MPK acquisition costs were offset by lower costs in other areas, such as housekeeping assistance and productivity loss.^23^

Given that previous economic evaluation studies were conducted outside the Netherlands and their results cannot be translated to the Dutch healthcare system, we performed an economic evaluation of MPKs and NMPKs in the Netherlands. The objectives of this study were to calculate the incremental cost-utility ratio and incremental cost-effectiveness ratio from a societal perspective and assess the relation of the incremental cost-utility ratio to the Dutch willingness-to-pay threshold.^24^ Based on previous studies,^18-21,25^ we hypothesized that the MPK would be cost-effective compared to the NMPK.

## METHODOLOGY

The Dutch guideline for the conduct of economic evaluations in healthcare was applied.^17^ Results are presented in accordance with the Consolidated Health Economic Evaluation Reporting Standards (CHEERS) statement.^26^

The Medical Ethics Committee of the University Medical Center Groningen (METc 2019/419) provided a waiver for formal approval. Research was conducted according to the Declaration of Helsinki and its amendments.

All participants were asked to provide their written informed consent before completing the survey. This study was registered at Clinicaltrials.gov: NCT06105944.

### Data Collection and Analyses

• ***Participants***

Individuals with a unilateral transfemoral amputation or knee-disarticulation, who were using a prosthesis, were eligible for participation. The inclusion criteria were: (1) at least 18 years old; (2) at least one year post amputation; (3) able to read and write in Dutch; (4) using a prosthesis with socket. Participants were recruited via two large prosthetic companies with multiple branches in the Netherlands.

• ***Data Collection***

Postal surveys were sent to eligible participants between December 2022 and March 2023. Non-respondents received a reminder after 12 weeks. Participants received a €10 gift voucher for their participation.

Study data were collected and managed using REDCap electronic data capture tools.^27,28^ Participants who did not meet the inclusion criteria, did not state their prosthetic knee type or did not complete the EQ-5D-5L were excluded from analyses.

### Survey Development

The survey consisted of a questionnaire with four separate sections: 1) patient demographics; 2) costs related to LLP use; 3) health-related QoL, and 4) prosthesis-related QoL.

• ***Patient Demographics***

Participants provided information on their age, sex, educational level, employment status, the side and level of their limb loss, the type of prosthetic knee they were currently using, and their years of prosthesis experience.

• ***Costs Related to Prosthesis Use***

To assess medical consumption and productivity costs, we combined the iMTA Medical Consumption Questionnaire (iMCQ)^29^ and the iMTA Productivity Cost Questionnaire (iPCQ).^30^ The iMCQ measures medical consumption, household assistance, and help from friends and family, while the iPCQ evaluates productivity losses, including absenteeism, presenteeism, and unpaid work.^31^ To tailor the questionnaires to LLP users, we replaced questions about dieticians, speech therapists and emergency room visits with questions about prosthesis type and visits to a rehabilitation facility or certified prosthetist/orthotist (CPO). Furthermore, we added questions about personal costs for prosthesis acquisition, repairs, home or vehicle adjustments and hobbies. This resulted in a 24-items questionnaire (**Appendix I**). Psychometric properties of this questionnaire are not available.

While no validity studies have been conducted yet, the majority of the questions within the iPCQ were sourced from existing validated questionnaires, with the exception of the section addressing “productivity losses related to unpaid work”^31^ Moreover, the development of the iMCQ took place in the Netherlands, tailoring it to the country's healthcare system.^29^

The recall periods of the iMCQ and iPCQ were extended to six months to capture a reliable overview of LLP-related costs. The recall period defines the time span participants should reflect on when answering the questionnaire items. Direct medical costs, informal care, and travel expenses were derived from the questionnaires, while indirect medical costs related to productivity loss were calculated using the friction cost method with a friction period of 115 days, including the value of unpaid work.^17^ The friction cost method estimates the economic impact of productivity losses due to illness or premature death by considering only the time required to replace a worker and restore production, rather than the entire period of absence. Costs for appointments with healthcare providers were valued at standard Dutch prices,^32^ and costs for an appointment with the CPO were based on average outpatient consultation costs.^32^ Acquisition costs for NMPK and MPK were based on information derived from orthopaedic workshops, and presenteeism and absenteeism were determined according to Dutch guidelines.^32^

• ***Health-Related Quality of Life***

Participants completed the Dutch version of the EuroQol - five dimensions - five levels (EQ-5D-5L),^33,34^ a self-assessment tool with five questions on mobility, self-care, daily activities, pain, and anxiety/depression. Each question has five response levels, defining a unique health state. The Dutch scoring algorithm for the EQ-5D-5L was used to compute a single value representing health status. Scores can range from -0.466 to 1, with a higher score representing a better QoL.^35^ Participants also rated their perceived health on a visual analogue scale (VAS) from 0 (worst imaginable health) to 100 (best imaginable health). The EQ-5D-5L is a reliable and valid questionnaire,^36^ with satisfactory measurement properties for patients with major unilateral LLA.^37^

• ***Prosthesis-Related Quality of Life***

The utility, ambulation and well-being scales of the Prosthesis Evaluation Questionnaire (PEQ) were used, as they align with EQ-5D-5L items. The PEQ is a reliable and valid self-report tool for evaluating prosthesis-related QoL.^38^ It includes nine scales, as well as several separate questions which can be used independently.^39^ Questions are scored on a VAS (0–100), with higher scores indicating more positive outcomes.

### Health Economic Evaluation

Two methods are commonly used for an economic evaluation: 1) a cost-utility analysis or 2) a cost-effectiveness analysis. In a cost-utility analysis, the effects are expressed in quality-adjusted life years (QALYs). QALYs are calculated by adjusting life years for a utility measure reflecting quality, ranging from 0 (death) to 1 (full health).^16^ In this study, the utility score of the EQ-5D-5L was multiplied with the six-month measurement period to calculate the QALYs. Comparing the difference in QALYs to the difference in costs, results in the incremental cost-utility ratio (ICUR), or cost per QALY gained^40^ (see equations below). The value of this parameter can be compared across different diseases and also to the threshold value for willingness-to-pay for a gain of one QALY. The values of these willingness-to-pay thresholds vary across countries and are linked to the burden of disease (higher burden of disease equals a higher threshold). In contrast, a cost-effectiveness analysis uses a clinical effect measure to calculate the incremental cost-effectiveness ratio (ICER), representing the additional cost per unit of a specific health outcome, such as improved mobility or prosthesis-related QoL (see equation below). Unlike the cost-utility analysis, which uses QALYs as a generic measure, a cost-effectiveness analysis can focus on specific, relevant outcomes for prosthesis users. This allows for a more detailed understanding of how cost differences between prosthetic knees can impact prosthesis users' daily lives.



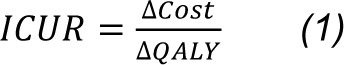




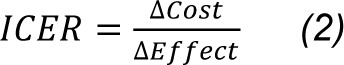


ICUR: incremental cost utility ratio; ICER: incremental cost effectiveness ratio; ∆: difference.

The economic evaluation in this study was based on cross-sectional data, adopting a societal perspective and including direct medical costs and indirect costs. The ICUR was calculated by dividing the mean cost difference between NMPK and MPK by the mean QALY difference based on the EQ-5D-5L. Furthermore, three ICERs were calculated by dividing the mean cost difference by the mean score difference on the PEQ scales.

To estimate statistical uncertainty and robustness of results, we used the bootstrap method to simulate 5000 repetitions of the study, with variations in mean incremental costs and effects.^41^ Bootstrap results are presented as a scatterplot in a cost-effectiveness plane (CE-plane) with incremental effects on the x-axis and incremental costs on the y-axis. The CE-plane is divided into the north-east (NE) quadrant, the north-west (NW) quadrant, the south-west (SW) quadrant and the south-east (SE) quadrant. Replications in these quadrants represent the following results: NE (better health outcomes, higher cost), NW (worse health outcomes, higher cost), SE (better health outcomes, lower cost), and SW (worse health outcomes, lower cost). The bootstrap results were used to construct a cost-effectiveness acceptability curve (CEAC) summarizing the probability of cost-effectiveness of the MPK over the NMPK at various willingness-to-pay thresholds for each QALY gained. The value of this threshold depends on the burden of disease (**Appendix II - Table 1A**). A specific burden of disease score was not available for LLA or prosthesis use and was therefore calculated based on the Dutch guidelines^24^ (**Appendix II - Table 2A**).

### Statistical Analyses

To address missing data, we applied multiple imputation and bootstrapping. Multiple imputation was used to generate several complete datasets by replacing missing values with plausible estimates based on observed data patterns. Bootstrapping was then performed on these imputed datasets to assess the stability and variability of the results, providing more reliable statistical inference while accounting for uncertainty introduced by the missing values. Missing values for CPO visits and informal care (11 and 3 cases, respectively) were imputed using average numbers. Prior to bootstrap replication, missing data for healthcare visits (10 cases) were handled by multiple imputation, adjusted for age, sex, prosthesis type, amputation level, and prosthesis side. The average of 50 imputations was used for the bootstrap procedure.

Continuous variables were assessed for normality and variance equality using Q-Q plots, Kolmogorov-Smirnov tests, and Levene's tests. Differences in demographics, EQ-5D-5L utility and VAS scores, PEQ scale scores, and costs between groups of prosthesis users were evaluated using Kruskal-Wallis tests, Mann-Whitney U tests or unpaired t-tests for continuous variables, and a Pearson's χ2 test for categorical variables. All tests were two-tailed with significance set at p<0.05. Since none of the continuous variables met the assumptions of a one-way ANOVA, only Kruskal-Wallis tests were performed, followed by Mann-Whitney U tests with Bonferroni correction (p<0.005). Data analyses were conducted using IBM SPSS Statistics version 28 (IBM Corporation, Armonk, NY, USA) and Stata version 18 SE (StataCorp, College Station, TX, USA).

## RESULTS

**Figure 1:**
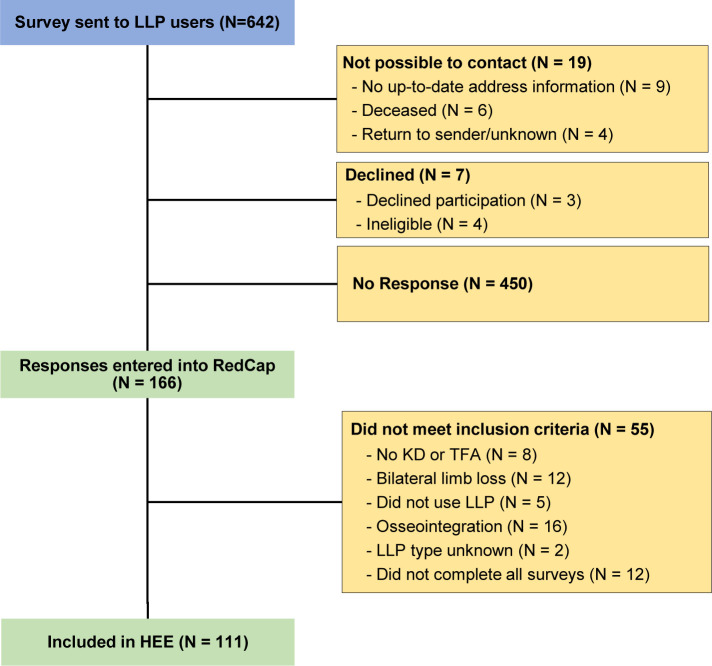
Flowchart of survey distribution (blue), response (yellow), and inclusion process (green) for the health economic evaluation (HEE). **LLP:** lower limb prosthesis; **KD:** knee-disarticulation; **TFA:** transfemoral amputation.

Surveys were sent to 642 participants, with 166 responding (response rate 28.5%) (**Figure 1**). Forty-one respondents did not meet the inclusion criteria. Additionally, the knee type was unknown for two respondents and twelve respondents did not complete all parts of the survey. Ultimately, 111 respondents were included (age 64 ± 13 years; **Table 1**). MPK users were significantly younger and the time since amputation was significantly lower compared to NMPK users. Furthermore, we found significant differences in whether participants were using their first prosthesis and if they had obtained a new prosthesis within the last six months, with the NMPK group scoring higher in both scenarios.

**Table 1:** Demographic characteristics of 111 participants categorized by type of prosthetic knee.

	NMPK (n = 49)	MPK (n = 62)	p-value
**Age, (Mean ± SD)**	67 ± 12	62 ± 14	0.027^*^
**Sex, n (%)^a^**			0.953^†^
• Female	16 (33)	21 (34)	
• Male	32 (65)	41 (66)	
**Side of LLA, n (%)^a^**			0.063
• Left	17 (35)	33 (53)	
• Right	31 (63)	29 (47)	
**Level of LLA, n (%)^a^**			0.417
• Transfemoral	33 (67)	38 (61)	
• Knee-Disarticulation	15 (31)	24 (39)	
**Employment Status, n (%)^a^**			0.246
• Wage Employment	5 (10)	15 (24)	
• Self-Employed	6 (12)	7 (11)	
• Homemaker	6 (12)	5 (8)	
• Unemployed	1 (2)	0	
• Incapacity to Work	4 (8)	9 (15)	
• Retired	27 (55)	26 (42)	
**Years Since Amputation, (Mean ± SD)**	28 ± 24	16 ± 18	0.005^*^
**Level of Education^a,b^**			0.324
• Low	21 (43)	18 (29)	
• Middle	15 (30)	25 (40)	
• High	13 (27)	18 (29)	
**New Prosthesis in Last 6 Months, yes n (%)**	10 (20)	10 (16)	0.029^*^
**First Prosthesis, yes n (%)**	13 (27)	3 (5)	0.001^*^

**NMPK:** non-microprocessor-controlled knee; **MPK:** microprocessor-controlled knee; **SD**: standard deviation; **LLA**: lower limb amputation

a Some variables have missing responses and therefore do not add up to 100%

b **Low:** no education or lower vocational education; **Middle:** middle vocational education; **High:** higher education such as university of applied sciences or university (BSc/MSc)

* Significant at α < 0.05.

† This p-value indicates that there was no significant difference in the gender distribution between the two groups.

### Costs Related to Prosthesis

All cost components, including visits to healthcare professionals, prosthesis acquisition, and productivity losses, were valued using Dutch standard pricing and data obtained from orthopaedic workshops and national guidelines (**Table 2**).

**Table 2:** Unit costs used in calculations.

Unit	Price/distance	Source/remark
**Hospital Admission**	€644.00	Guideline, no distinction general/university hospital
**Outpatient Visits**		Dutch guidelines
• CPO	€120.00	Guideline average price outpatient visit
• Rehabilitation	€120.00	Guideline average price outpatient visit
**Visits Healthcare Professionals**		
• General Practitioner	€30.87	Guideline, per visit
• Physiotherapist	€38.89	Guideline, per visit
• Occupational Therapist	€24.32	Guideline, per visit
• Social Worker	€127.00	Guideline, per visit
• Psychologist/Psychiatrist	€109.80	Guideline, average primary care and private
• Occupational Health Physician	€200.00	Average price Occupational Health and Safety service
**Household Support and Informal Care**		
• Household Help	€32.76	Guideline, per hour
• Personal Care	€57.58	Guideline, per hour
• Nursing Care	€75.00	Guideline, per hour
• Informal Care	€18.80	Guideline, per hour
**Out Of Pocket Costs**	Real costs	Guideline
**Paid Work**		
• Friction Period (Days)	115	Guideline, average past 5 years
• Friction Period (Weeks)	16.4	Guideline, average past 5 years
• Productivity Cost/Hour	€39.88	Average for male and female, per hour
**Travel Costs**		Dutch guidelines
• Car	€0.26	Euro/km, parking costs € 3.00 per visit
• Public Transport	€0.21	Euro/km
• Taxi	€2.47	Euro/km, start costs € 3.36 per ride
• Unknown	€0.26	Euro/km, car price applied
**Average Travel Distances**		
• General Practitioner	1.1 km	Guideline
• Physiotherapist	2.2 km	Guideline
• Occupational Therapist	2.2 km	Assumed same as physiotherapist
• Social Worker	7.0 km	Assumed same as hospital
• Psychologist/Psychiatrist	7.0 km	Assumed same as hospital
• Occupational Health Physician	3.7 km	Average travel distance home to work
• Hospital	7.0 km	Guideline
**Prosthetic Knee Costs**		
• MPK	€21,018	Expert opinion
• NMPK	€4,417	Expert opinion

### Mean Medical and Non-Medical Costs

Mean medical and non-medical costs were categorized into six groups: hospital admission, CPO visits, visits to other healthcare professionals (HCP), productivity loss, other costs, and total costs. No significant differences in mean costs between groups were demonstrated in hospital admission, CPO visits, visits to other HCPs, combined costs for visits to HCPs and productivity loss (**Table 3**). In ‘other costs’, significantly higher costs for household care (p = 0.016) were found in the NMPK group compared to the MPK group. However, total costs were significantly higher for the MPK group (p < 0.001) mainly due to higher acquisition costs (**Table 3**). More detailed information is shown in **Appendix III - Table A3**.

**Table 3:** Mean costs (in Euros) by category and type of prosthetic knee.

	NMPK (N = 42) ^#^	MPK (N = 55)	p-value
**Hospital admission**			
• Direct Costs	**184** (0, 0–7728)	**46** (0, 0–1932)	0.754
**Outpatient visits**			
• CPO	**357** (142, 0–2148)	**302** (249, 0–1351)	0.491
**Other HCP visits**			
• General Practitioner	**290** (0, 0–10605)	**38** (0, 0–331)	0.921
• Physiotherapist	**130** (0, 0–1381)	**335** (0, 0–2298)	0.106
• Occupational Therapist	**4** (0, 0–84)	**3** (0, 0–140)	0.887
• Social Worker	**6** (0, 0–268)	**12** (0, 0–669)	0.849
• Psychologist/Psychiatrist	**3** (0, 0–117)	**26** (0, 0–886)	0.445
• Occupational Health Physician	**24** (0, 0–817)	**4** (0, 0–206)	0.400
• Combined HCP Visit Costs	**458** (17, 0–10988)	**418** (0, 0–2419)	0.081
**Other costs**			
• Out of Pocket Costs	**1094** (0, 0–20000)	**1420** (0, 0–43000)	0.678
• Household Care	**1678** (0, 0–25657)	**270** (0, 0–3407)	0.016*
• Informal Care	**822** (0, 0–13686)	**1190** (0, 0–17597)	0.473
**Productivity loss**			
• Friction Costs	**368** (0, 0–15442)	**161** (0, 0–4786)	0.483
• Presenteeism	**21** (0, 0–383)	**101** (0, 0–3988)	0.769
**Total costs**			
• Prosthesis (Fixed)	**4417**	**21018**	NA
• Total Costs, Excluding Prosthesis	**4981** (1308; 0–39340)	**3909** (1376; 0–43108)	0.730
• Total Costs, Including Prosthesis	**9395** (5725, 4417–43757)	**24927** (22394, 21018–64126)	<0.001*

Data presented as mean (median, min-max). Significance was tested using the Mann Whitney U test.

**NMPK:** non-microprocessor controlled knee; **MPK:** microprocessor controlled knee; **CPO:** certified prosthetist/orthotist; **NA:** not applicable.

* Significant at α < 0.05.

# In tables presenting statistical results, the number of participants depends on the available complete data.

### Outcome Measures

A significant difference in the PEQ ambulation scale was observed between the NMPK and MPK. No significant differences were found on the remaining PEQ scales, as well as the EQ-5D-5L utility score and VAS score (**Table 4**).

**Table 4:** Scores EQ-5D-5L and PEQ.

	NMPK (N = 49)	MPK (N = 60)*	Mean difference	p-value
**EQ-5D-5L utility**	0.742	0.787	0.045 (-0.028 to 0.118)	0.225
**EQ-5D-5L VAS**	73.5	78.3	4.78 (-3.58 to 13.1)	0.259
**PEQ-AM**	52.0	67.0	15.0 (-6.0 to 24.0)	0.001
**PEQ-UT**	69.2	74.0	4.8 (-1.8 to 11.5)	0.154
**PEQ-WB**	72.8	76.3	3.4 (-4.7 to 11.5)	0.402

**VAS:** visual analogue scale; **PEQ-AM**: prosthesis evaluation questionnaire ambulation scale; **PEQ-UT:** prosthesis evaluation questionnaire utility scale; **PEQ-WB:** prosthesis evaluation questionnaire well-being scale;

* N=58 for EQ-5D-5L

### Cost-Utility Analysis

**Figure 2:**
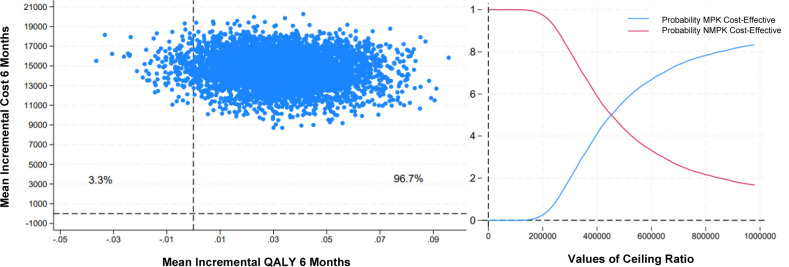
Left panel: Cost-effectiveness plane (CE-plane) showing the distribution of bootstrap replications for the differences between costs and effects of the MPK versus the NMPK. Blue dots indicate how many of the 5000 replications fall in the respective quadrants. The CE-plane is divided into the north-east (NE) quadrant, the north-west (NW) quadrant, the south-west (SW) quadrant and the south-east (SE) quadrant. Replications in these quadrants represent the following results: NE (better health outcomes, higher cost), NW (worse health outcomes, higher cost), SE (better health outcomes, lower cost), and SW (worse health outcomes, lower cost). Right panel: Cost-effectiveness acceptability curve (CEAC) showing the probability of cost-effectiveness of the MPK and NMPK at increasing values of the ceiling ratio for willingness-to-pay for a QALY gained.

The mean cost difference after bootstrap was €14,626, with lower costs for the NMPK, and the mean QALY difference was 0.032 in favor of the MPK. This resulted in a mean incremental cost-utility ratio (ICUR) of €457,063 per QALY gained (**Table 5**). Most bootstrap replications fell within the NE quadrant (**Figure 2, left panel**), indicating higher utility and higher costs for the MPK compared to the NMPK. The cost-effectiveness acceptability curve in **Figure 2** (**right panel**) demonstrates that the probability of the MPK being cost-effective does not exceed that of the NMPK until well over €400,000 per QALY, far above all willingness-to-pay thresholds (€20,000; €50,000 and €80,000).^24^

**Table 5:** ICUR and ICER calculation.

	NMPK (N = 46)	MPK (N = 55)*	P-value	Mean difference^†^	ICUR/ICER^‡^
**QALY**	0.37	0.40	0.08	0.03 (-0.04 to 0.09)	457,063
**PEQ-AM**	53.3	67.9	0.002	14.6 (1.1 to 30.5)	1,020
**PEQ-UT**	69.4	74.9	0.11	5.4 (-7.7 to 16.7)	2,757
**PEQ-WB**	72.8	77.4	0.27	4.5 (-10.7 to 18.5)	3,308

**ICUR:** incremental cost-utility ratio; **ICER:** incremental cost-effectiveness ratio; **NA:** not applicable; **PEQ:** prosthesis evaluation questionnaire; **AM:** ambulation; **UT:** utility; **WB:** well being;

* N=52 for QALY;

† Mean difference after bootstrap;

‡ ICUR/ICER calculated with mean cost difference of €14,626 after bootstrap; ICUR/ICER were calculated based on population that completed the PEQ/EQ5D and had costs.

### Cost-Effectiveness Analysis

**Figure 3:**
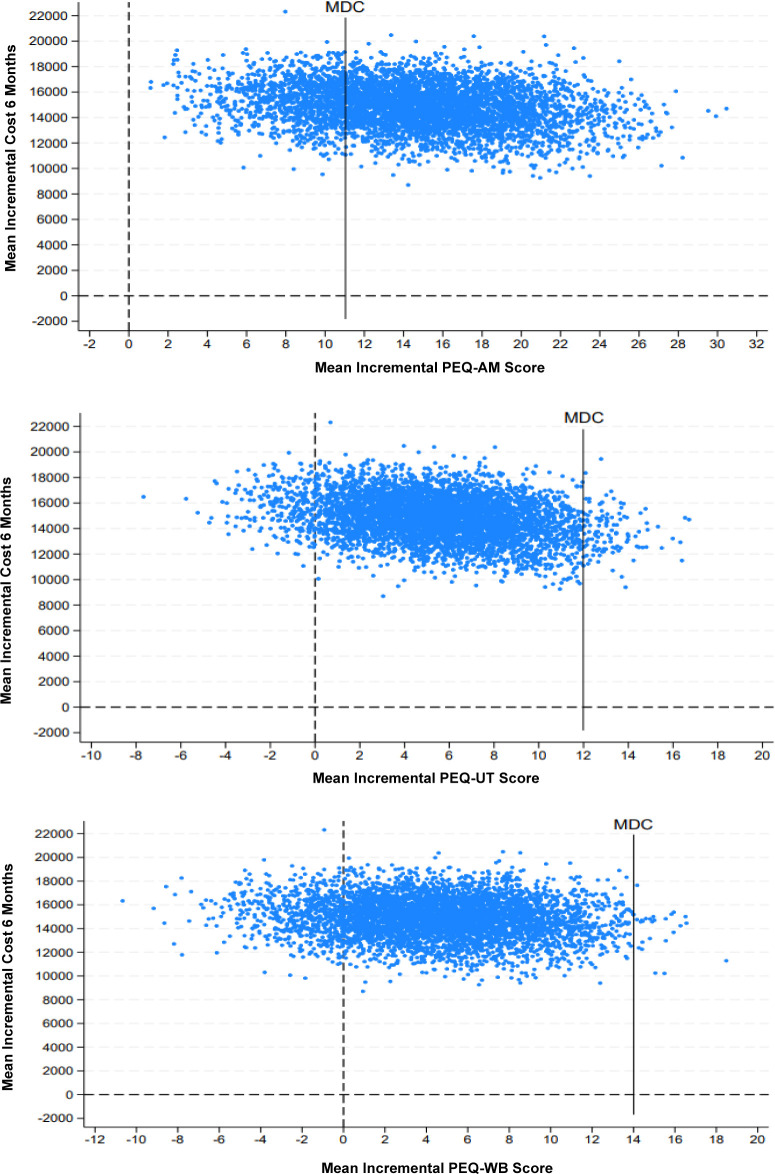
Cost-effectiveness planes (CE-planes) showing the distribution of bootstrap replications for the differences between costs and effects of the MPK versus the NMPK on the PEQ ambulation scale (top panel); PEQ utility scale (middle panel); PEQ well-being scale (bottom panel). MDC: minimal detectable change. The CE-plane is divided into the north-east (NE) quadrant, the north-west (NW) quadrant, the south-west (SW) quadrant and the south-east (SE) quadrant. Replications in these quadrants represent the following results: NE (better health outcomes, higher cost), NW (worse health outcomes, higher cost), SE (better health outcomes, lower cost), and SW (worse health outcomes, lower cost).

The mean score for the PEQ-ambulation scale after bootstrap was 14.6 points higher for the MPK group compared to the NMPK group, resulting in a mean incremental cost-effectiveness ratio (ICER) of €1,020 per point gained (**Table 5**). Nearly all bootstrap replications fell within the NE quadrant, indicating higher self-reported walking ability and higher costs for the MPK compared to the NMPK (**Figure 3-Top**). Furthermore, 78.1% of bootstrap replications exceeded the minimal detectable change (MDC) of 11 points.^42^

After the bootstrap, the mean score for the PEQ-utility scale was 5.4 points higher for the MPK group compared to the NMPK group, yielding a mean ICER of €2,757 per point gained (**Table 5**). Most bootstrap replications fell within the NE quadrant, indicating higher utility and higher costs for the MPK compared to the NMPK (**Figure 3-Middle**). Of all the bootstrap replications, 2.2% exceeded the MDC of 12 points.^42^

Lastly, a mean score difference of 4.5 points higher for the MPK group compared to the NMPK group was found on the PEQ-well-being scale after bootstrapping, resulting in a mean ICER of €3,308 per point gained (**Table 5**). The majority of the bootstrap replications fell within the NE quadrant, indicating better reported well-being and higher costs for the MPK compared to the NMPK (**Figure 3-Bottom**). Half a percent of the bootstrap replications exceeded the MDC of 14.^42^

## DISCUSSION

This study demonstrates that the MPK is likely to provide higher scores of prosthesis-related and health-related QoL to its users compared to the NMPK, but at higher societal costs. The cost-effectiveness analyses yielded reasonable ICERs for prosthesis-related QoL ranging from €1,020 to €3,308, indicating that the costs to achieve a minimally detectable change is very reasonable and well within the willingness-to-pay threshold. However, based on the ICUR for health-related QoL, the MPK was determined not to be cost-effective. It is important to note that both the ICUR and ICER were based on short term measurements and as was shown in previous studies^18,20,21^ the ICUR will most likely be lower, making the MPK more cost-effective over a longer period of time.

The significantly higher costs for the MPK were mostly driven by the higher acquisition costs, while the combined costs for visits to healthcare professionals were lower for this group compared to the NMPK group. As was shown in a previous study, the higher acquisition costs for the MPK will be balanced out after 19 months due to the lower healthcare costs related to falls.^43^ For clinicians and policy makers, this would mean that the higher initial costs are an investment that will lead to lower long-term costs and significant functional improvements for the prosthesis users.

### Mean Costs and Medical Consumption

The significant difference in total costs between MPK and NMPK was mainly driven by the fixed high acquisition costs of MPK (€21,018) compared to NMPK (€4,417). This is similar to previous studies.^18,20,22^ However, a recent study demonstrated that while the MPK's acquisition costs exceeded those of the NMPK, this would offset after 19 months due to lowered healthcare costs related to falls.^43^ When excluding the acquisition costs, total costs were higher for NMPK (**Table 3**). Higher costs for the NMPK group were related to higher combined costs for visits to HCPs and higher costs for household care. While this study did not investigate the specific causes for visits to HCPs, one study demonstrated that the medical costs for fall-related incidents were 2.67 times higher for NMPK users compared to MPK users, which made up 46% of the total costs for this group.^22^ Previous studies have demonstrated that the use of an MPK can result in a significant decrease in falls compared to an NMPK, which could substantiate the findings^4,6,8,10,13,44^ Therefore, it would be valuable for future research to examine the specific fall-related costs in more detail, as this could provide further insight into the economic impact of the MPK compared to the NMPK.

Furthermore, MPK users demonstrated higher presenteeism and lower friction costs. These findings support earlier studies reporting decreased household costs and less productivity loss with MPK compared to the NMPK.^23^ Recently, a Dutch study demonstrated significant improvements in participation with MPK use compared to NMPK,^10^ which could explain the reduced need for household care and lower work absence.

### Outcome Measures

The MPK group showed significantly higher scores on the PEQ ambulation scale compared to the NMPK group. This aligns with other studies reporting significant improvements in walking ability with MPK, including walking distance, speed, and terrain navigation,^11,45^ as well as self-reported walking ability.^4,45^

No significant differences were found on both the utility score and VAS score of the EQ-5D-5L between the MPK and NMPK, which contrasts earlier findings.^20,21^ Additionally, no significant differences were found in the utility and well-being scales of the PEQ, which contradicts previous studies that reported significantly higher scores on these scales for MPK users.^10,12,45,46^ The disparity could stem from methodological differences, since participants in this study were assessed using their own prosthesis without an intervention, preventing direct comparison between the MPK and NMPK. Participants in both groups may have been generally content with their current prostheses, resulting in no significant differences.

### Cost-Utility Analysis

Based on the ICUR, the MPK was not cost-effective compared to NMPK. Most bootstrap replications indicated improved QoL with MPK, but costs were higher, exceeding all willingness-to-pay thresholds. Our mean ICUR was €457,063 per QALY gained, more than ten times higher than other studies, which reported ICURs ranging from €3,218^15^ to €40,155.^20^ One study stratified participants into three groups based on age at enrolment; age at first prosthesis; and years of experience using a prosthesis, yielding ICURs between €28,269 and €88,779,^20^ but even the highest ICUR was much lower than ours. Mean incremental cost differences in other studies ranged from €7,657 to €18,431,^18,20,21^ comparable to our €14,626. However, QALYs gained with the MPK in other studies were much higher (0.42 – 2.38) than our finding of 0.032.^18,20-22^

Methodological differences likely explain the variance in results. Firstly, the six-month timeframe in this study contrasts with other studies' 5-year,^20,21^ 8-year^18^ or even 25-year^22^ periods. Longer follow-up with sustained improvement of QoL with the MPK is likely to result in larger QoL differences compared to the NMPK. Simultaneously, higher acquisition costs of the MPK could be compensated by lower healthcare consumption if measured over a longer period of time.

Furthermore, not all studies included the same costs. Our study included acquisition, direct, and indirect medical costs and productivity loss, whereas other studies limited costs to acquisition and maintenance,^18^ did not specify costs for HCPs other than the general practitioner,^21^ or analyzed acquisition, maintenance, and transportation costs but did not specify them.^20^ The lack of specific cost insights in other studies prevents direct comparison to further clarify the ICUR difference.

### Cost-Effectiveness Analysis

The ICERs based on prosthesis-related outcomes indicated that the MPK could result in better QoL at acceptable costs per unit of improvement compared to the NMPK. However, formal thresholds for acceptability of these cost differences for a unit of improvement are not available, so this remains arbitrary. Although a direct comparison with a formal threshold is not possible, it is worth noting that due to the fairly low ICERs, the costs associated with achieving the MDCs for each PEQ scale can be considered reasonably priced. E.g. the costs to achieve the MDC of 11 points for the PEQ ambulation scale would cost €11,215. In this study, we demonstrated a significant difference in score on the PEQ-ambulation scale, which exceeded the MDC in 78.1% of the bootstrap replications. This is comparable to previous studies that investigated the differences in walking ability between the MPK and NMPK^4,12,21,45,47^ While we did not find significant differences in the other PEQ-scales, other studies using intervention designs with a within-subject comparison did report significant differences on these scales.^10,12,45,46^ This design difference may explain the variation in outcomes.

### Limitations

This study had several limitations. Firstly, we only considered the cost of the prosthetic knee unit, excluding other parts of the prosthesis such as the socket, pylon and foot, which may result in an underestimation of the total cost. Moreover, we were unable to obtain prosthesis costs at the individual level, leading to absence of variation in prosthesis cost as a major component of total costs. Additionally, prosthesis users differed between those using an NMPK or an MPK, with variations in age, activity level, and health status potentially influencing the outcomes and generalizability of the findings. Lastly, our non-intervention design did not allow for direct comparison between the NMPK and MPK.

## CONCLUSION

The cost-effectiveness analyses demonstrated that the MPK is likely to improve prosthesis-specific QoL, but at higher costs. However, short-term (6 months) improvement in health-related QoL was too small to result in substantial QALY gain to offset the higher costs of the MPK and result in an incremental cost-utility ratio below the generally accepted willingness-to-pay thresholds.

## Footnotes

* More recent cost data were not available due to inconsistencies in the registration of assistive devices by health insurance companies. As a result, the National Health Care Institute has restricted access to detailed datasets (email correspondence, November 2024).

## APPENDIX

### Appendix I English translation of the cost-questionnaire

The following questions are about your job. That means work for which you are paid. The questions refer to the job you held during the past 6 months. If you do not have a paid job, please continue with question 10. Be sure to read the explanation above question 10 first.

**Question 1. What was your occupation during the past 6 months?**

…………………………………………………………………………………………………………………..

**Question 2. How many hours per week did you work during the past 6 months?** Only count the hours for which you were paid. ………… hours

**Question 3. How many days per week did you work during the past 6 months?** ………… days

**Question 4. Have you been absent from work due to illness during the past 6 months?** *This refers to absence or sick leave related to your prosthesis or amputation*.

☐ No
☐ Yes, I was unable to work for the entire 6 months
☐ Yes, I was absent for approximately ….. days *(Only count the working days in the past 6 months)*

If you checked ‘’Yes,’’ please answer question 5. Otherwise, continue with question 7.

**Question 5. Were you absent from work for more than 4 consecutive weeks due to illness during the past 6 monts?**

☐ No
☐ Yes

If you checked ‘’Yes,’’ please answer question 6. Otherwise, continue with question 7.

**Question 6. How long were you ill for?** *This refers to absence or sick leave related to your prosthesis or amputation*. Approximately …… weeks

**Question 7. Were there days during the past 6 months when you worked but experienced physical or psychological problems during work?** *This refers to complaints related to your prosthesis or amputation*.

☐ No
☐ Yes

If you checked ‘’Yes,’’ please answer question 8 and 9. Otherwise, continue with question 10. *Please read the explanation above question 10 first*.

**Question 8. On how many working days did you experience physical of psychological problems during work?** Only count the working days in the past 6 months. Approximately …… working days

**Question 9. On the days you experienced problems, you may not have been able to work as much as usual. How much work could you do on those days on average?** *Refer to the scale below. 10 means you could work as much as usual. 0 means you could do nothing. Circle the appropriate number*.

**Explanation for question 10 and 11: Unpaid work**
You may also experience physical or psychological problems with unpaid work. Sometimes this means you can do less. For example, you may struggle to care for your children, do volunteer work, go grocery shopping, or work in the garden. The following questions are about this. Ook bij onbetaald werk kunt u last hebben van uw lichamelijke of psychische problemen. Soms kunt u daardoor minder doen. U kunt bijvoorbeeld niet goed voor de kinderen zorgen of vrijwilligerswerk doen. Of geen boodschappen doen of in de tuin werken. Daarover gaan de volgende vragen.

**Question 10. Were there days during the past 6 months when you could do less unpaid work due to physical or psychological problems?** This refers to problems related to your prosthesis use during the past 6 months.

☐ No
☐ Yes

If you checked ‘’Yes,’’ please answer question 11. Otherwise, continue with question 12.

**Question 11. On how many days did this occur?** Only count the days in the past 6 months …… days

**Explanation**
We would like to know which doctors you had appointments with during the past 6 months. These should be appointments for yourself. Other healthcare providers also count. For example, appointments with a physical therapist or CPO/prosthetist. Which appointments count?

- Check-ups
- Appointments due to physical or psychological complaints
- Home visits by a doctor
- Telephone appointments
- Calls to the prescription line

What appointments do not count?

- Appointments for someone else, such as your partner or child
- Calls to schedule an appointment

If you're not sure how many appointments you had, please write down an approximate number.

**Question 12. Have you been admitted to a rehabilitation center during the past 6 months?**

☐ No admission
☐ ….. days of admission

**Question 13. How many appointments did you have with your general practitioner or practice nurse during the past 6 months?**

☐ No appointments
☐ ….. appointments

**Question 14. How many appointments did you have with a social worker during the past 6 months?**

☐ No appointments
☐ ….. appointments

**Question 15. How many appointments did you have with you prosthetist/CPO?**

☐ No appointments
☐ ….. appointments

**Question 16. How many appointments did you have with a physical therapist during the past 6 months? Or with a Caesar therapist, Mensendieck therapist, or manual therapist?** Only count appointments outside of the hospital or rehabilitation center. Add all appointments with these therapists together.

☐ No appointments
☐ ….. appointments

**Question 17. How many appointments did you have with an occupational therapist during the past 6 months?** Only count appointments outside the hospital or rehabilitation center.

☐ No appointments
☐ ….. appointments

**Question 18. How many appointments did you have with a psychologist, psychotherapist or psychiatrist during the past 6 months?** Only count appointments outside of the hospital or rehabilitation center. Add all appointments with these professionals together

☐ No appointments
☐ ….. appointments

**Question 19. How many appointments did you have with an occupational health physician during the past 6 months?**

☐ No appointments
☐ ….. appointments

**Question 20a. Have you received home care during the past 6 months?** *This only refers to support or care received in connection with your prosthesis or amputation*.

☐ No
☐ Yes

If you checked ‘’Yes,’’ please answer question 20b through 20d. Otherwise, continue with question 21.

**Question 20b. What type of home care did you receive during the past 6 months?**

☐ Household help
  *e.g., vacuuming, making the bed, grocery shopping*
☐ Personal care
  *e.g., help with showering or dressing*
☐ Nursing care
  *e.g., applying bandages, administering medication, measuring blood pressure*

**Question 20c. How many weeks did you receive this home care?** Add up all weeks in the past 6 months. Note: a 6-month period equals 26 weeks.

**Table:**

Household help:	….. weeks
Personal care:	….. weeks
Nursing care:	….. weeks

**Question 20d. How many hours of home care did you receive on average during these weeks?**

**Table:**

Household help:	Average ….. hours per week
Personal care:	Average ….. hours per week
Nursing care:	Average ….. hours per week

**Question 21a. Have you received help from a family member or acquaintance during the past 6 months due to physical or psychological problems?** *This refers only to help received in connection with your prosthesis or amputation*.

☐ No
☐ Yes

If you checked ‘’Yes,’’ please answer question 21b through 21d. Otherwise, continue with question 22.

**Question 21b. What type of help did you receive from family members or acquaintances during the past 6 months?** *You may check more than one box*

☐ Household help
  *e.g., vacuuming, making the bed, grocery shopping, preparing food and drinks, caring for children*
☐ Personal care
  *e.g., help with showering or dressing, help with eating and drinking, administering medication*
☐ Practical help
  *e.g., support with walking, outings or visits to acquaintances, visits to the doctor or hospital, arranging help or financial matters*

**Question 21c. How many weeks did you receive this home care?** Add up all weeks in the past 6 months. Note: a 6-month period equals 26 weeks.

**Table:**

Household help:	….. weeks
Personal care:	….. weeks
Practical help:	….. weeks

**Question 21d. How many hours of home care did you receive on average during these weeks?**

**Table:**

Household help:	Average ….. hours per week
Personal care:	Average ….. hours per week
Practical help:	Average ….. hours per week

**Question 22. Have you or your family members spent extra money in the past 6 months on any of the following items?** *These expenses are related to your prosthesis or amputation*

**Table:**

Category	No	Yes	Estimated amount (€)
Repairs of the prosthesis at own expense			€
Home modifications at own expense			€
Assistive devices at own expense			€
Modifications to vehicles at own expense (car, bike, motorcycle)			€
Equipment for hobbies/sports at own expense			€
Other, namely ……………………			€

**Explanation**
The following questions are about expenses incurred in connection with visits to the hospital, rehabilitation center and CPO/prosthetist.

**Question 23. What means of transportation did you use to travel from home to the hospital, rehabilitation center, or CPO/prosthetist?**

☐ Not applicable
☐ Walking
☐ Bicycle
☐ Car
☐ Wheelchair or mobility scooter
☐ Public transport
☐ Taxi
☐ Other, namely ………………………………………………………………………

**Question 24. What is the one-way distance between your home and the hospital, rehabilitation center, and CPO/prosthetist?**

**Table:**

Hospital:	….. kilometer
Rehabilitation center:	….. kilometer
CPO/prosthetist:	….. kilometer

**Do you have any questions or comments?**

If you have any questions or comments, please write them down below.

……………………………………………………………….…………………………………

……………………………………………………………….…………………………………

……………………………………………………………….…………………………………

……………………………………………………………….…………………………………

……………………………………………………………….…………………………………

……………………………………………………………….…………………………………

### Appendix II Calculation of burden of disease

The severity of an illness or condition can be expressed in a burden of disease score between 0 and 1. The different willingness-to-pay thresholds (WTP) are based on this score (Table A1).^24^

**Table A1:** Willingness-to-pay thresholds in the Netherlands.

Burden of disease	Reference threshold for maximum additional cost per QALY
0,1 – 0,4	Up to €20,000 per QALY
0,41 – 0,7	Up to €50,000 per QALY
0,71 – 1,0	Up to €80,000 per QALY

*QALY: quality adjusted life year*

The burden of disease is calculated with the following equation:

Remaining QALYs in a specific health state are calculated by multiplying the EQ-5D-5L index score by the remaining number of years in that health state.

To assess the burden of disease associated with lower limb amputation (LLA), we compared the health-adjusted life expectancy between the general population in the Netherlands and persons with peripheral vascular disease (PVD), the primary underlying condition in this study population.^48^ The average life expectancy of the general population is 85.5 years,^49^ while individuals with PVD have an estimated life expectancy of 81.5 years.^50^

The average EQ-5D-5L index score for the general population of the Netherlands was 0.869^35^ which is in line with the index scores of comparable countries that ranged from 0.82 to 0.90.^51-54^ For the LLA population, EQ-5D-5L index scores ranged from 0.462 to 0.531.^37^

The average age at amputation was estimated at 67 years, based on available cohort data.

Based on these parameters, we calculated the burden of disease across three scenarios, yielding a range of 0.48 to 0.60 (Table A2). Given that the range of burden of disease falls within the Dutch WTP of €50,000 for a burden of disease between 0.41 and 0.70, this threshold is considered appropriate for evaluating interventions in the LLA population.

**Table A2:** Burden of disease scores.

		Burden of disease score
1	Smallest possible difference in EQ-5D-5L index score	0,48
2	Largest possible difference in EQ-5D-5L index score	0,60
3	Best fitting variables for LLA population	0,50

### Appendix III

**Table A3:** Detailed costs (in Euros) and volumes by category and knee type.

	NMPK (N=44)	MPK (N=58)
**Outpatient visits**		
*CPO*		
Direct costs (€)	253.6 (n=29)	263.2 (n=45)
Travel costs (km)	93.2 (n=28)	51.7 (n=42)
**Other HCP visits**		
*Direct costs (€)*		
General practitioner	74.4 (n=19)	36.2 (n=25)
Physiotherapist	99.8 (n=13)	293.0 (n=26)
Occupational therapist	2.76 (n=2)	2.52 (n=2)
Social worker	5.77 (n=1)	https://doi.org/10.9 (n=1)
Psychologist/psychiatrist	2.50 (n=1)	20.8 (n=3)
Occupational health physician	22.7 (n=2)	3.44 (n=1)
*Travel costs combined (€)*	205.2 (n=22)	58.6 (n=37)
**Other costs**		
Out of pocket costs (€)	1047 (n=17)	1407 (n=25)
*Household care (h)*		
Household work	1434 (n=13)	461.5 (n=6)
Personal care	704 (n=4)	n=0
Nursing care	n=0	n=0
Informal care (h)	2357 (n=14)	3591 (n=16)
**Productivity loss**		
Friction costs (€)	524.8 (n=1)	226.7 (n=3)
Presenteeism (€)	21.9 (n=4)	142.3 (n=3)

**NMPK:** non-microprocessor controlled knee; **MPK:** microprocessor controlled knee; **CPO:** certified prosthetist/orthotist
